# *MKK4* variants rs3826392 and rs3809728 are associated with susceptibility and clinicopathological features in colorectal cancer patients

**DOI:** 10.22038/ijbms.2021.56874.12690

**Published:** 2021-08

**Authors:** Kimberly Carol Esmeralda Martínez-Casillas, Anilú Margarita Saucedo-Sariñana, Patricio Barros-Núñez, Martha Patricia Gallegos-Arreola, Tomás Daniel Pineda-Razo, María Eugenia Marín-Contreras, Silvia Esperanza Flores-Martínez, Mónica Alejandra Rosales-Reynoso

**Affiliations:** 1 División de Medicina Molecular, Centro de Investigación Biomédica de Occidente, Instituto Mexicano del Seguro Social (IMSS), Guadalajara, Jalisco, México; 2 Unidad de Investigación Seguimiento Enfermedades Metabólicas, Unidad Médica de Alta Especialidad Pediatría, Instituto Mexicano del Seguro Social (IMSS), Guadalajara, Jalisco. México; 3 División de Genética, Centro de Investigación Biomédica de Occidente, Instituto Mexicano del Seguro Social (IMSS), Guadalajara, Jalisco, México; 4 Servicio de Oncología Médica, Hospital de Especialidades, Instituto Mexicano del Seguro Social (IMSS), Guadalajara, Jalisco, México; 5 Servicio de Gastroenterología, Hospital de Especialidades, Instituto Mexicano del Seguro Social (IMSS), Guadalajara, Jalisco, México

**Keywords:** Colorectal cancer, Haplotypes, MKK4, Susceptibility, Variants

## Abstract

**Objective(s):**

The mitogen-activated protein kinase kinase 4 (*MKK4*) plays a key role in several processes like inflammation, apoptosis, and tumorigenesis. Several authors have proposed that genetic variations in these genes may alter their expression with subsequent cancer risk. This study aimed to examine the possible association of *MKK4* rs3826392 and rs3809728 variants in Mexican patients with colorectal cancer (CRC). These variants were also compared with clinical features as sex, age, TNM stage, and tumor location.

**Materials and Methods:**

The study included genomic DNA from 218 control subjects and 250 patients. Genotyping of the *MKK4* variants was performed using polymerase chain reaction-restriction fragment length polymorphism (PCR-RFLP) procedure.

**Results:**

Individuals with A/T and T/T genotypes for the rs3809728 (-1044 A>T) variant showed a significantly increased risk for CRC (*P=*0.012 and 0.007, respectively); while individuals with the G/G genotype for the rs3826392 (-1304 T>G) variant showed a decreased risk for CRC (*P=*0.012). Genotypes of the MKK4 rs3809728 variant were also significantly related to colon localization and advanced TNM stage in CRC patients. T-T haplotype (rs3826392 and rs3809728) of the *MKK4* gene was associated with risk in patients with CRC.

**Conclusion:**

The rs3826392 variant in the *MKK4* gene could be a cancer protective factor, while the rs3809728 variant could be a risk factor. These variants play a significant role in CRC risk.

## Introduction

Colorectal cancer (CRC) is one of the most prevalent cancers and the second cause of death worldwide (1, 2). In Mexico, the incidence and mortality rate in 2020 were 11.6/100,000 and 6/100,000 inhabitants, respectively (3). Genetic and environmental factors give individuals CRC risk (4-6). Studies on epidemiology of sporadic CRC have determined several etiologic factors related to individual lifestyles, such as alcohol and tobacco consumption, sex, body mass index, high fat diet, and processed and red meat (7-12). These factors are known as cellular stressors, and their signals are transduced by the mitogen-activated protein kinase (MAPK) pathways leading to inflammation, apoptosis, and tumorigenesis (13, 14).  

Mitogen-activated protein kinase kinase 4 (MKK4) is a member of the stress-activated protein kinase (SAPK) signaling pathway. The SAPK pathway is composed of several kinases working in sequential steps (MAPK, MAPKK, and MAPKKK, respectively) (15, 16). This pathway is triggered by several stimuli such as environmental stresses, inflammatory cytokines, and growth factors (16, 17). The *MKK4* gene is situated in chromosome 17p11.2 and contains 11 exons (18, 19). The MKK4 protein is an essential part of the MAPK signaling pathway involved as a central mediator of the c-JUN NH2-terminal kinase cascade (JNK), which in turn is the main link to the RAS oncogenic signaling pathway (18, 20). *MKK4* activates the kinases c-Jun NH2-terminal (JNK) and p38 (21) and induces several biological responses due to stress stimuli, hormones, pro-inflammatory cytokines, and growth factors (21-24).

MKK4 participates actively in the processes of apoptosis, cell differentiation, and gene transcription (23, 25-29). It has been described that overexpression of *AXIN1/2* negatively regulates the Wnt pathway and leads to differential activation of MKK4 and MKK7, which play essential roles in cell growth and tumorigenesis (30). *MKK4* has been identified as a metastasis suppressor gene in prostate and ovarian cancers (31, 32). Likewise, the lack of expression of the *MKK4* gene has been associated with poor survival in gastric adenocarcinoma (18). 

The effect of* MKK4* variants on cancer susceptibility has been assessed in CRC (28, 33), lung cancer (24), acute myeloid leukemia (34), nasopharyngeal carcinoma (35), cervical cancer (36), and breast cancer (37). Among them, the rs3826392 (-1304 T>G) variant is sited in the promoter of the *MKK4* gene and has been frequently shown as a protective genetic factor in several cancers, including CRC (24, 28, 34, 35, 37); however, the variant rs3809728 (-1044A>T), also located in the *MKK4* promoter, has not been associated with any cancer (24, 28, 34, 35). 

This study assesses for the first time, possible association of their genotypes, alleles, and haplotypes for the -1304 T>G (rs3826392) and -1044 A>T (rs3809728) variants of the *MKK4* gene with the development and clinicopathological features of CRC.

## Materials and Methods

***Study population***

Four hundred sixty-eight individuals with diagnosis of colorectal adenocarcinoma were included as stated by the anatomopathological criteria of the Specialty Hospital of the Mexican Institute of Social Security (IMSS) in Guadalajara, Mexico. The CRC stage was established according to the TNM classification. The patients group consisted of 250 patients (117 females and 133 males) and the control group consisted of 218 healthy subjects (121 females and 97 males), which were not matched by age with the patients group. All individuals included in this study came from the Guadalajara metropolitan area, Mexico. The study was approved by the Ethical Committee 1305 (R-2018-1305-001) of the IMSS and was conducted following the national and international ethical standards. The samples were taken after signed informed consent. We utilized an epidemiologic questionnaire to collect personal information for all individuals and the clinical and pathological characteristics of patients were taken from the hospital records. 

***Genotyping analysis***

DNA samples were obtained according to Miller´s method (38). The variants rs3826392 (-1304T>G) and rs3809728 (-1044A>T) in the *MKK4* gene were analyzed by polymerase chain reaction-restriction fragment length polymorphism (PCR-RFLP) using previously described primers (25). The PCR reaction for both variants (rs3826392 and rs3809728) was performed in a total volume of 10 μl which contained: 1 X PCR buffer (100 mM Tris- HCl, 500 mM KCl, and 0.1% Triton TMX-100), 2.0 mM MgCl2, 150 μM dNTPs, 1 μM of each primer, 2 U Platinum Taq DNA Polymerase, and 100 ng DNA. The program of PCR carried out in a thermocycler was as follows: activation of the Platinum Taq DNA polymerase enzyme 5 min at 95 ^°^C, followed by 35 cycles of denaturation at 94 ^°^C for 45 sec, annealing (at 58 ^°^C for variant rs3826392 and 60 ^°^C for the variant rs3809728) for 45 sec and extension at 72 ^°^C for 45 sec, with a final extension step at 72 ^°^C for 7 min. 

PCR amplification products were corroborated by electrophoresis on 6% polyacrylamide gels, then 5 μl of the DNA amplified by PCR were digested with 4 units of the MluCI restriction enzyme (New England Biolabs, USA) at 65 ^°^C overnight for the rs3809728 (-1044 A>T) variant, and for the rs3826392 (-1304 T>G) variant with the AflII restriction endonuclease (New England Biolabs, USA) at 37 ^°^C overnight. 

**Figure 1:**
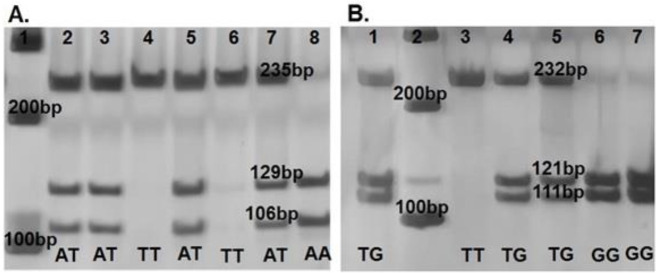
Genotype representation of *MKK4* variants (rs3809728 and rs3826392) on 6% polyacrylamide gels stained with AgNO3

The digestion fragments were separated on 6% polyacrylamide gels. The assignation of the genotypes was realized according to fragments of digestion generated by cutting site of the restriction enzymes. For analysis of the rs3809728 (-1044 A>T) variant the MluCI enzyme recognizes the sequence ^AATT and cuts before the adenine nucleotide. In samples with genotype homozygous A/A (wild type) the fragments of digestion were 129 and 106 bp. In samples with genotype A/T (heterozygous) the fragments 235, 129, and 106 were observed, and in the samples with genotype homozygous T/T (polymorphic) only one fragment of 235 bp was observed, because this does not have the site of restriction for the MluCI enzyme. To corroborate the results, randomly 10% of the samples were re-genotyped using another method and the result was 100% concordant (Figure 1A).

For the assignation of genotypes of the rs3826392 (-1304 T>G) variant, the AflII enzyme recognizes the site C^TTAAG and cuts after cytosine nucleotide. In the samples with genotype homozygous T/T (wild type) only one fragment of 232 bp was observed, because this does not have the site of restriction for the AflII enzyme. In the samples with genotype T/G (heterozygous), the fragments observed were 232, 121, and 111 bp, and in samples with genotype homozygous G/G (polymorphic) the fragments were 121 and 111 bp in length (Figure 1B).

***Statistical analysis***

Genotype and allele frequencies were determined by direct counting in the groups. To determinate Hardy-Weinberg equilibrium (HWE) and to evaluate categorical variables the Chi-square test was performed. In the analysis of association of genotypes and alleles with clinical and anatomopathological characteristics, the odds ratio (OR) with confidence intervals of 95% (CI) and Chi-square with Yates´s correction was performed in SPSS 25.0 software package (SPSS Inc., Chicago, IL, USA) and Epi info 6. The analysis of haplotypes was realized in Haploview 4.2. A *P-*value<0.05 was considered statistically significant. 

## Results

***Demographic and clinicopathological features in the study groups***

All analyzed samples of control and CRC groups were efficiently genotyped for *MKK4* rs3826392 (-1304 T>G) and rs3809728 (-1044A>T) variants. Table 1 shows the demographic, clinical, and pathological data of subjects included in the study. We observed statistical differences regarding age distribution (*P*=0.001). The CRC individuals showed an age range of 29 to 59 years; while for the healthy individuals, the age range was of 27 to 59 years. The consumption of tobacco and drink showed statistical significance between the two groups. Concerning the clinicopathological features of the patients with CRC: 69.2% had stage III-IV tumors, 32% had metastases, and 46.8% had tumors located in the rectum.

***MKK4 variants in patients and control subjects***

The *MKK4* variants in study groups showed statistical significance (Table 2). In the healthy group, the SNPs analyzed were in Hardy-Weinberg equilibrium (HWE) (*P*>0.05). For the rs3826392 variant, the HWE was X_2_=1.55 (*P*=0.21); and for rs3809728 variant the HWE was X_2_=0.73 (*P*=0.39). The genotype G/G of the *MKK4* rs3826392 variant (-1304T>G) was found in 24.4% (61/250) of the CRC patients and in 35.8% (78/218) of the healthy group; this difference shown statistical significance (OR=0.46; 95% CI=0.26–0.82, *P*=0.013). Allelic frequency differences were also statistically significant; G allele carriers has a protective effect for CRC (OR=0.70; 95% CI=0.54-0.91, *P*=0.009). 

Concerning the *MKK4* rs3809728 variant (-1044A>T), the patients and the control group individuals exhibited significant differences between A/T and T/T genotypes (*P*=0.012 and *P*=0.007, respectively). Under a dominant pattern (A/T+T/T vs A/A) it showed that the allele T is associated with CRC risk (OR=2.58; 95% CI=1.32-5.03, *P*=0.006). Likewise, allele frequencies analysis showed that the allele T is associated with susceptibility to CRC (OR=1.33; 95% CI=1.01-1.74, *P*=0.043).

***MKK4 genotypes by sex and age***

Table 3 shows analysis of the *MKK4* genotypes regarding sex and age. Decreased risk was observed in CRC female patients in presence of the G/G genotype for the rs3826392 variant (OR=0.35; 95% CI=0.15-0.81, *P*=0.023); in addition, a marginal association was observed for females with CRC regarding TG+GG dominant model (OR=0.46; 95% CI=0.22-0.96, *P*=0.057). Regarding age, patients over 50 years and carrying the G/G genotype showed a protective effect (OR=0.21; 95% CI=0.07-0.66, *P*=0.010). 

For the rs3809728 variant we observed that the male patients carrying A/T and TT genotypes showed an increased risk (OR=4.12; 95% CI=1.64-10.32, *P*=0.003 and OR=3.50; 95% CI=1.39-8.77, *P*=0.010, respectively); and this association was observed with the dominant model (OR=3.80; 95% CI=1.58-9.11, *P*=0.003). Regarding the age, we did not observe a statistical significance.

***MKK4 genotypes by anatomopathological features***

Association of *MKK4* genotypes with TNM stages and tumor site are shown in Tables 4 and 5. Analysis adjustment by age showed that the patients with presence of G/G genotype for the rs3826392 variant have a protective effect for TNM III+IV stages (OR=0.45; 95% CI=0.23-0.87, *P*=0.027); while, individuals with the T/T genotype for the rs3809728 variant, have a marginally significant difference for TNM III+IV stages (OR=2.28; 95% CI=1.03- 5.03, *P*=0.058) (Table 4). In contrast, in the analysis by tumor site adjusted by age; we observed an increased risk for developing tumors in colon in patients with A/T and T/T genotypes for the rs3809728 variant (OR=4.43; 95% CI=1.28-15.24, *P*=0.019 and OR=4.35; 95% CI=1.24-15.21, *P*=0.025, respectively) (Table 5). 

***MKK4 Haplotypes***

The haplotype analysis showed that the combination T-T of rs3826392 (-1304 T>G) and rs3809728 (-1044 A>T) alleles in the *MKK4* gene increased the risk for developing CRC (OR=1.82; 95% CI=1.19-2.79, *P*=0.007) (Table 6).

A. *MKK4* rs3809728 (-1044A>T) variant. Lane 1: 100 bp molecular marker. Lanes 2, 3, 5, 7: Individuals with genotype heterozygous (A/T) showing bands of 235+129+106 bp. Lanes 4 and 6: Individuals with genotype homozygous polymorphic (T/T) showing band of 235 bp. Lane 8: Individual with genotype homozygous wildtype (A/A) showing bands of 129+106 bp

B. *MKK4* rs3826392 (-1304T>G) variant. Lanes 1, 4, 5: Individuals with genotype heterozygous (T/G) showing bands of 232+121+111 bp. Lane 2: 100 bp molecular marker. Lane 3: Individual with genotype homozygous wildtype (T/T) showing band of 232 bp. Lanes 6, 7: Individuals with genotype homozygous polymorphic (G/G) showing bands of 121+111 bp

**Table 1:** Demographic and clinical characteristics in colorectal cancer patients and control subjects

Characteristics	CRC group n= 250 (100%)	Control group n= 218 (100%)	*P*-value
Mean Age (years ± SD)	58.68 (±59.5)	37.94 (±11.74)	0.001
Age 50 years			
<50	46 (18.4)	173 (79.3)	0.001
>50	204 (81.6)	45 (20.7)	
Sex			
Female Male	117 (46.8) 133 (52.3)	121 (55.5) 97 (44.5)	0.060
Smoking status			
Yes No	87 (34.8) 163 (65.2)	30 (13.8) 188 (86.2)	0.001
Drinking status			
Yes No	73 (29.2) 176 (70.4)	27 (12.4) 191 (87.6)	0.001
Diabetes mellitus Yes No	70 (28.0) 180 (72.0)	75 (34.4) 143 (65.6)	0.143
Hypertension			
Yes No	84 (33.6) 166 (66.4)	71 (32.5) 147 (67.5)	0.813
**Clinical stage TNM **			
I II III IV	7 (2.8) 70 (28) 93 (37.2) 80 (32)		
**Tumor site**			
Colon Rectum NA	108 (43.2) 117 (46.8) 25 (10)		

*P* *values* were calculated by the Chi-square test

CRC: colorectal cancer; NA: Not available

**Table 2:** Distribution of genotypes and allelic frequencies of *MKK4* polymorphisms and colorectal cancer risk

	** Frequencies **			***p ***
**Genotype**	**CRC group n=250 (%)**	**Control group n=218 (%)**	**OR (95% CI)**	***P-*** **value**
***MKK4 *** **(rs3826392)**				
**T/T**	47 (18.8)	28 (12.8)	1.00 (Reference)	
**T/G**	142 (56.8)	112 (51.4)	0.75 (0.44-1.28)	0.364
**G/G**	61 (24.4)	78 (35.8)	**0.46 (0.26-0.82)**	**0.013**
**T/G+G/G vs. T/T**	203 (81.2)	190 (87.1)	0.63 (0.38-1.05)	0.104
**Allele**				
**T**	236 (47.2)	168 (38.5)	1.00 (Reference)	
**G**	264 (52.8)	268 (61.5)	**0.70 (0.54-0.91)**	**0.009**
***MKK4 *** **(rs3809728)**				
**A/A**	14 (5.6)	29 (13.3)	1.00 (Reference)	
**A/T**	131 (52.4)	109 (50.0)	**2.48 (1.25-4.94)**	**0.012**
**T/T**	105 (42.0)	80 (36.7)	**2.71 (1.34-5.48)**	**0.007**
**A/T+T/T vs. A/A**	236 (94.4)	189 (86.7)	**2.58 (1.32-5.03)**	**0.006**
**Allele**				+
**A **	159 (31.8)	167 (38.3)	1.00 (Reference)	
**T**	341 (68.2)	269 (61.7)	**1.33 (1.01-1.74)**	**0.043**

CRC: Colorectal cancer; Bold text highlights statistically significant findings; *P-values* were calculated by the chi-square test

**Table 3:** Association of *MKK4* polymorphisms with sex and age in CRC patients and controls

**rs3826392 (-1304 T>G)**						
	**Patients/Control**			** OR (95% CI); ** ***P*** **-value**		
**V** **ariable**	**TT**	**TG**	**GG**	**TG versus TT**	**GG versus TT**	**TG+GG versus TT**
Sex						
Male	23/15	76/45	34/37	1.10 (0.52-2.32); 0.950	0.59 (0.26-1.33); 0.290	0.87 (0.42-1.78); 0.849
Female	24/13	66/67	27/41	0.53 (0.25-1.13); 0.145	**0.35 (0.15-0.81); 0.023**	**0.46 (0.22-0.96); 0.057**
Age (years)						
<50	10/23	30/86	6/64	0.80 (0.34-1.87); 0.775	**0.21 (0.07-0.66); 0.010**	0.55 (0.24-1.26); 0.233
>50	37/5	112/26	55/14	0.58 (0.20-1.62); 0.418	0.53 (0.17-1.59); 0.380	0.56 (0.20-1.52); 0.357
**rs3809728 (-1044 A>T)**						
	**Patients/Control**			** OR (95% CI); ** ***P*** **-value**		
**Variable**	**AA**	**AT**	**TT**	**AT versus AA**	**TT versus AA**	**AT+TT versus AA**
Sex						
Male	8/19	66/38	59/40	**4.12 (1.64-10.3); 0.003**	**3.50 (1.39-8.77); 0.010**	**3.80 (1.58-9.11); 0.003**
Female	6/10	65/71	46/40	1.52 (0.52-4.43); 0.605	1.91 (0.63-5.74); 0.366	1.66 (0.58-4.74); 0.479
Age (years)						
<50	1/23	26/88	19/62	6.79 (0.87-52.7); 0.070	7.04 (0.89-55.6); 0.069	6.90 (0.90-52.5); 0.060
>50	13/6	105/21	86/18	2.30 (0.78-6.76); 0.214	2.20 (0.73-6.57); 0.259	2.26 (0.80-6.31); 0.199

Bold text highlights statistically significant findings

**Table 4:** Association between TNM stage and *MKK4* rs3826392 and rs3809728 polymorphisms in CRC patients and controls

**rs3826392 (-1304 T>G)**													
** Genotype**	**TNM Stage** **I+II** **n=77 (%)**	**TNM stage** **III+IV ** **n=173 (%)**	**Control** **n=218** **(%)**	**I+II stage ** ***vs *** **control ** **OR (95% CI)**	***P-*** **value**		**I+II stage ** ***vs *** **control ** **OR (95% CI) ** *	***P*** -value*		**III+IV stage ** ***vs *** **control ** **OR (95% CI)**	***P-*** **value**	**III+IV stage ** ***vs *** **control ** **OR (95% CI) ** *	***P-*** value*
**T/T**	14 (18.2)	33 (19.1)	28 (12.8)	1.00 (Reference)		1.00 (Reference)			1.00 (Reference)			1.00 (Reference)	
**T/G**	42 (54.5)	100 (57.8)	112 (51.4)	0.75 (0.36-1.56)	0.563	1.03 (0.42-2.47)		1.000	0.75 (0.42-1.34)		0.418	0.68 (0.37-1.23)	0.262
**G/G**	21 (27.3)	40 (23.1)	78 (35.8)	0.53 (0.24-1.20)	0.189	0.80 (0.31-2.06)		0.840	**0.43 (0.23-0.81)**		**0.014**	**0.45 (0.23-0.87)**	**0.027**
**T/G+G/G**	63 (81.2)	140 (80.9)	190 (87.1)	0.66 (0.32-1.33)	0.335	0.93 (0.40-2.18)		1.000	0.62 (0.36-1.08)		0.122	0.58 (0.33-1.04)	0.091
**rs3809728 (-1044A>T)**													
**Genotype**	**TNM stage I+II ** **n= 77 (%)**	**TNM stage III+IV n=173 (%) **	**Control** ** n=218** **(%)**	**I+II stage ** ***vs *** **control ** **OR (95% CI)**	***P-*** **value**		**I+II stage ** ***vs *** **control ** **OR (95% CI) ** *	***P*** -value*		**III+IV stage ** ***vs *** **control ** **OR (95% CI)**	***P-*** **value**	**III+IV stage ** ***vs *** **control ** **OR (95% CI) ** *	***P-*** value*
**A/A **	4 (5.2)	10 (5.8)	29 (13.3)	1.00 (Reference)		1.00 (Reference)			1.00 (Reference)			1.00 (Reference)	
**A/T **	42 (54.5)	89 (51.4)	109 (50.0)	2.79 (0.92-7.42)	0.096	2.92 (0.83-10.22)		0.131	**2.36 (1.09-5.12)**		**0.039**	1.91 (0.87-4.17)	0.140
**T/T**	31 (40.3)	74 (42.8)	80 (36.7)	2.80 (0.91-8.65)	0.103	2.77 (0.77-9.95)		0.171	**2.68 (1.22-5.88)**		**0.019**	**2.28 (1.03-5.03)**	**0.058**
**A/T+T/T **	73 (94.8)	163 (94.2)	189 (86.7)	2.80 (0.95-8.24)	0.083	2.86 (0.84-9.75)		0.127	**2.50 (1.18-5.28)**		**0.021**	2.07 (0.97-4.39)	0.078

*Adjust for age in >50 years. Bold text highlights statistically significant findings

**Table 5:** Association between tumor location and polymorphisms rs3826392 and rs3809728 of *MKK4 *in CRC patients and controls

**rs3826392 (-1304 T>G)**											
**Genotype**	**Colon cancer** **n=108 (%)**	**Rectal cancer** **n=117 (%)**	**Control ** **n= 218 (%)**	**Colon cancer vs control** **OR (95% CI)**	***P-*** **value**	**Colon cancer vs control** OR (95% CI) *	***P-*** value *	**Rectal cancer ** **vs ** **control** **OR (95% CI)**	***P-*** **value**	**Rectal cancer vs control** OR (95% CI)*	***P-*** value*
**T/T**	20 (18.5)	24 (20.5)	28 (12.8)	1. 00 (Reference)		1.00 (Reference)		1.00 (Reference)		1.00 (Reference)	
**T/G**	60 (55.5)	66 (56.4)	112 (51.4)	0.75 (0.39-1.44)	0.487	0.63 (0.32-1.26)	0.267	0.68 (0.36-1.28)	0.308	0.79 (0.39-1.60)	0.647
**G/G**	28 (26.0)	27 (23.1)	78 (35.8)	0.50 (0.24-1.03)	0.088	0.49 (0.23-1.04)	0.098	**0.40 (0.20-0.81)**	**0.016**	0.53 (0.25-1.15)	0.163
**T/G+G/G **	88 (81.5)	93 (79.5)	190 (87.1)	0.64 (0.34-1.24)	0.232	**0.54 (2.66-12.66)**	**0.008**	0.57 (0.31-1.03)	0.091	0.69 (0.35-1.34)	0.363
**rs3809728 (-1044 A>T)**											
**Genotype**	**Colon cancer** **n=108 (%)**	**Rectal cancer** **n=117 (%)**	**Control n=218 (%)**	**Colon cancer vs control** **OR (95% CI)**	***P-*** **value**	**Colon cancer vs control** OR (95% CI) *	***P-*** value *	**Rectal cancer ** **vs ** **control** **OR (95% CI)**	***P-*** **value**	**Rectal cancer vs control** OR (95% CI)*	***P-*** value*
**A/A**	3 (2.8)	9 (7.7)	29 (13.3)	1. 00 (Reference)		1.00 (Reference)		1.00 (Reference)		1.00 (Reference)	
**A/T**	60 (55.5)	59 (50.5)	109 (50.0)	**5.32 (1.55-18.20)**	**0.006**	**4.43 (1.28-15.24)**	**0.019**	1.74 (0.77-3.92)	0.244	1.43 (0.60-3.37)	0.539
**T/T**	45 (41.7)	49 (41.8)	80 (36.7)	**5.43 (1.56-18.85)**	**0.006**	**4.35 (1.24-15.21)**	**0.025**	1.97 (0.86-4.51)	0.151	1.81 (0.75-4.32)	0.251
**A/T+T/T**	105 (97.2)	108 (92.3)	189 (86.7)	**5.37 (1.59-18.05)**	**0.004**	**4.39 (1.30-14.83)**	**0.017**	1.84 (0.84-4.03)	0.172	1.59 (0.69-3.62)	0.356

*Adjust for age in > 50 years. Bold text highlights statistically significant findings

**Table 6:** Haplotype analysis in the *MKK4* gene in CRC patients

**Haplotype**		** Frequencies**		**X** ^2^	**CRC Risk** **OR (95% CI)**	*** P*** **-value**
***MKK4 *** **gene**		**CRC group **N**= **250 **(%)**	**Control group **N**= **218 **(%)**			
**rs3826392- ** **rs3809728**						
**G**	**T**	91 (36.6)	90 (41.3)	0.974	0.81 (0.56-1.18)	0.323
**T**	**T**	79 (31.6)	44 (20.4)	7.255	**1.82 (1.19-2.79)**	**0.007**
**G**	**A**	41 (16.2)	44 (20.2)	0.881	0.77 (0.48-1.24)	0.347
**T**	**A**	39 (15.6)	40 (18.1)	0.446	0.82 (0.50-1.33)	0.504

CRC: colorectal cancer; Bold text highlights statistically significant findings

## Discussion

Previous investigations have pinpointed a considerable number of gene mutations associated with CRC; however, some results are controversial regarding the potential effect of the *MKK4* gene on CRC susceptibility. Specifically, the rs3826392 (-1304 T>G) variant has been related to a protective effect on CRC development in the Chinese population (26); meanwhile, the rs3809728 (-1044 A>T) variant, although frequently studied, has not been associated with CRC. With this background, the objective of this study was to evaluate two SNVs (rs3826392 and rs3809728) of the *MKK4* gene and their effects on CRC. Our results suggest that rs3826392 and rs3809728 variants of the *MKK4* gene participate in the development of CRC in the Mexican population studied here.

Among the 250 analyzed patients, we observed a significant increase in CRC in people over 50 years (81.6%), which is consistent with the results of several previous studies (39-41). The American Society of Clinical Oncology (ASCO) in 2019 indicated that the average age at the time of colon cancer diagnosis is approximately 70 years, while, for rectal cancer, the average age is approximately 63 years. Although CRC can occur at younger ages, its risk increases preponderantly in people over 50 years of age. 

In this study, cancer risk was evident in individuals with A/T and T/T genotypes for the rs3809728 variant; such a finding had not been previously reported in patients with different cancers analyzed, including CRC (24, 28, 34, 35, 37). Meanwhile, a protective effect was observed among patients carrying the G/G genotype of the rs3826392 (-1304 T>G) variant. 

Studies have shown that the polymorphic G allele of the rs3826392 variant enhances the transcriptional activity of the *MKK4* gene compared with the wild type T allele, suggesting that the -1304T>G change may increase the *MKK4* gene expression. As a member of the MAPK signaling pathway, the tumorigenic role of the *MKK4* protein is complex. Confirmation of the *MKK4* gene as a tumor suppressor has been obtained from different cancer cell lines in which, loss of heterozygosity (LOH) or presence of missense variants, produce a loss of function of *MKK4* (23, 27, 34).This loss of function due to mutations or decreased expression of *MKK4* has been described in patients with biliary cancer (42).

On the other hand, studies in breast and pancreas cancer demonstrate that *MKK4* has a pro-oncogenic activity (42, 43). Overexpression of the* MKK4* gene has been observed in gastric and prostate cancers (18, 44). A bioinformatic analysis realized by Wei *et al*. in 2009 showed that the T allele of the rs3826392 variant has a binding site for the transcription factor Nkx-2 (28), which plays an oncogenic role in several cancers like prostate, lung, Ewing´s sarcoma, and neuroendocrine tumors (28, 45-48). They also demonstrated that this allele increases the expression of *MKK4.* Therefore, it is plausible to assume that Nkx-2 may inhibit *MKK4* expression and lead to carcinogenesis in CRC tissue; however, for the rs3809728 A>T variant, no difference in binding factors was observed in the Wei *et al*. report (28). 

In our study, haplotype analysis showed that the T wild type allele in the rs3826392 (1304T>G) variant and the polymorphic T allele of the rs3809728 (-1044A>T) variant (haplotype T-T) is also associated with increased susceptibility to CRC.

Regarding age and sex of the patients studied here, females under 50 years old and carrying the G/G genotype for the rs3826392 variant showed a significantly decreased risk. This decreased risk was also found in 2009 by Wei *et al*. in Chinese patients with sporadic CRC and other types of cancer (28). Meanwhile, male patients with presence of A/T and T/T genotypes for the rs3809728 variant have increased CRC susceptibility. Such an observation has not been previously reported.

On the other hand, in the TNM stage and tumor site evaluation, our data suggest that patients over 50 and with A/T and T/T genotypes for the rs3809728 variant have an increased risk to reach advanced TNM stages (TNM III+IV). This susceptibility, which means a poorer prognosis in these patients, is probably related to unknown mechanisms that would allow a faster tumor progression. Inversely, the decreased risk observed in patients over 50 years with the rs3826392 risk variant is related to a better prognosis in these patients, possibly related to a slower tumor progression. 

Regarding tumor location, we observed that patients aged over 50 years had added susceptibility to develop colon cancer in the presence of the A/T and T/T genotypes for the rs3809728 variant. In contrast, for the rs3826392 variant, the patients aged over 50 years had a protective effect on rectum cancer in the presence of the G/G genotype. In support of these results, several clinical and biological features indicate that colon cancer is different from rectum cancer and these differences are related to embryological origin, function, anatomy, genetics, clinical manifestation, treatment response, and clinical outcome (49-54), and consequently, the therapies for rectal and colon cancer are also distinct, depending on the TNM stage (55). In studies realized in the Mexican population, patients with CRC also showed a greater predisposition to develop tumors in the colon (56, 57).

## Conclusion

As previously reported, our results showed that the *MKK4* rs3826392 variant operates as a protective factor for CRC; however, for the first time, these results also reveal that the rs3809728 variant is associated with an increased risk of CRC. Some genotypes of rs3826392 and rs3809728 variants are related to the TNM stage and tumor site in these patients. Interestingly, an association of the T-T haplotype (rs3826392 and rs3809728 alleles) with CRC risk was also demonstrated in this analysis. Further studies, including analysis of these variants in larger samples and functional studies, are necessary to confirm our results. Nevertheless, it is acceptable to suggest that the *MKK4* rs3826392 and rs3809728 variants can be considered useful biomarkers of prognosis and tumor site in CRC.
